# The impact of vascular access type on survival in haemodialysis: time for a paradigm shift? A prospective cohort study

**DOI:** 10.1007/s40620-023-01675-z

**Published:** 2023-08-01

**Authors:** Maria Paparella, Matthias Cassia, Rossella De Leonardis, Mario Cozzolino

**Affiliations:** 1 Department of Health Sciences, University of Milan, Milan, Italy; 2 Ospedale San Carlo Borromeo, ASST Santi Paolo e Carlo, Milan, Italy

**Keywords:** Haemodialysis, Vascular access, Tunnelled central venous catheter, Elderly

## Abstract

**Introduction:**

Although arteriovenous autologous fistula is the vascular access of choice due to better long-term outcome than central venous catheters, the use of central venous catheters is increasing. Our study aims to describe the survival and epidemiological features of a cohort of dialysis patients with a focus on the role of vascular access.

**Methods:**

Our study comprises a follow-up period from 2001 to 2020 in a single center. Descriptive analysis was performed on baseline data. Moreover, we analysed predictive variables of death with univariable and multivariable logistic regressions. Predictors of survival were analysed by univariable and multivariable Cox regression.

**Results:**

Our analysis includes 754 patients undergoing chronic haemodialysis. In the multivariable logistic regression, the use of tunnelled catheters resulted protective against death from any cause (Odds Ratio 0.43; *p* = 0.017). In the multivariable Cox analysis, being “late referral” was associated with decreased survival in the first 6 months since haemodialysis start (Hazard Ratio 3.79; *p* = 0.001). In the subgroup of elderly (age ≥ 75 years) patients (*n* = 201/472) with a follow up of 7–60 months, multivariable logistic regression showed that tunnelled catheters at the start of haemodialysis were associated with lower mortality (Odds Ratio, 0.25; *p* = 0.021), whereas vascular disease was found to be the main risk factor for death (Odds Ratio, 5.11; *p* = 0.000). Moreover, vascular disease was confirmed as the only independent risk factor by Cox analysis (Hazard Ratio, 1.58; *p* = 0.017).

**Conclusions:**

In our cohort, mortality was found to be more closely associated with comorbidities than with the type of vascular access. Tunnelled central venous catheters might be a viable option for haemodialysis patients.

**Graphical abstract:**

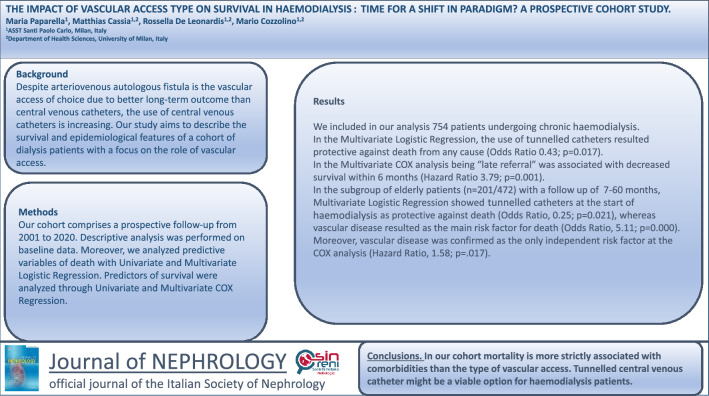

## Introduction

Over the past decades, several studies have investigated the impact of comorbidities and vascular access type on the mortality rate of haemodialysis patients [1–4]. This is particularly relevant among the elderly, as age further complicates the clinical picture. In the vascular access field, several data derived from national registries highlighted the increase in survival using arteriovenous fistulas over other vascular accesses, such as arteriovenous grafts or central venous catheters. This eventually led to the 2006 ‘’Fistula First’’ Breakthrough Initiative, sponsored by the Kidney Disease Outcomes Quality Initiative Vascular Access guidelines, aiming to promote the use of arteriovenous fistulas as the first choice of vascular access [5–7]. Despite a steep increase in their use, a significant proportion of patients still initiate dialysis with central venous catheters due to several reasons, such as unscheduled or urgent start of renal replacement therapy, the presence of severe vascular disease impairing proper maturation of the fistula, or an expected duration of dialysis less than one year [1, 8, 9]. These observations led the ‘‘Fistula First’’ Initiative, later renamed ‘‘Fistula first, Catheter last’’ Initiative [10], to reconsider and accept the use of catheters in particular circumstances. More recent data [7, 11–14] questioned the strength of the association between vascular access type and patient mortality, being highly influenced by confounding factors; this led to the statement of the 2019 vascular access guidelines [15] ‘‘the right access, in the right patient, at the right time, for the right reasons”, opening to the personalization of vascular access and the use of catheters in subgroups of patients [16, 17].

In this study, we aimed to investigate epidemiological features and survival of our cohort of 754 incident patients, exploring the role of comorbidities and type of vascular access in predicting mortality.

## Methods

All patients that started maintenance haemodialysis in our centre were enrolled prospectively from 1st January, 2001 to 30th November, 2020. Maintenance haemodialysis was defined as a renal replacement treatment continued for at least 1 month. Patients that started haemodialysis temporarily due to acute kidney injury, that shifted from peritoneal dialysis, or that failed renal transplantation were excluded from the study. Patients were also excluded if transferred to our centre having started haemodialysis elsewhere. Collected data included demographics, causes of end-stage kidney disease, clinically or histologically determined if biopsy was available, type of vascular access at the start of haemodialysis (arteriovenous fistula, arteriovenous graft, jugular or femoral temporary and tunnelled catheters), comorbidities at the start of the treatment identified clinically as defined by international standards. In particular, we defined severe cardiomyopathy if the patient was symptomatic with New York Heart Association class ≥ 3 at baseline; vascular disease was defined as radiologic evidence or direct clinical complications of atherosclerotic disease within any peripheral, central or cerebral vessels. Among the causes of end-stage kidney disease, we defined “renovascular” as any macro or microvascular disease affecting the kidneys. The elderly population was defined as being 75 years of age or older. The outcome of interest was death from any cause. Patients were followed until death, change of renal replacement modality (peritoneal dialysis, kidney transplantation) or loss to follow-up (data censoring).

### Statistical analysis

Statistical analysis was performed with Statistical Package for the Social Science (SPSS), version 23.0. Categorical data are presented as percentages, whereas continuous variables as means (± SD) or medians for skewed data. Categorical variables were compared with the chi-square test, while the two-tailed *t*-test for unpaired samples or one-way ANOVA, as appropriate, were used for continuous variables. Predictors of death from any cause were estimated as unadjusted Odds Ratio (OR) with 95% confidence interval using simple logistic regression analysis. Multivariate logistic regression analysis was used to check for confounding variables estimating adjusted OR with 95% confidence interval; stepwise forward selection was applied (enter limit and remove limit *P* < 0.05 and *P* < 0.10, respectively). Univariate survival analysis was generated according to the Kaplan–Meier method, and time-to-event curves for groups were compared using the log-rank test. Evidence for violation of proportional hazards assumption was not found; Cox proportional hazard regression model was applied with univariate analysis to calculate unadjusted Hazard Ratio (HR) with 95% Confidence Interval for each variable and multivariate analysis to correct for confounding factors (adjusted HR). Stepwise forward selection (enter limit and remove limit *P* < 0.05 and *P* < 0.10, respectively) was used. All statistical tests were two-tailed, *P* value of less than 0.05 was considered to indicate statistical significance.

## Results

### Baseline Characteristics

In our cohort of 754 patients (515 male, 239 female) aged 68 ± 14 years old, 40% (303/754) were defined as elderly (age ≥ 75 years); the incident rate of elderly increased from 17% in 2001 to 42% in 2020. Among the primary causes of end-stage kidney disease (Table 1), renovascular causes were the most common, accounting for 24% of all causes, followed by diabetic nephropathy, which affected 16% (117/754) of the patients. Vascular disease at the beginning of haemodialysis affected 60% (452/754) of patients, of whom 240/452 (53%) were aged ≤ 74 years old. At the start of haemodialysis, 35% (254/754) of patients had a functional arteriovenous fistula, whereas 57% (429/754) and 7% (56/754) initiated renal replacement treatment with a temporary and tunnelled catheter, respectively (Table 1). A second vascular access was finalized in 68% (516/754) of patients within 1.7 ± 1.3 months after the start of dialysis. At the end of the follow-up period, the prevalence of fistulas, tunnelled catheters, grafts and temporary catheters changed to 52% (391/754), 34% (256/754), 4.6% (34/754) and 10% (73/754), respectively.

**Table 1:** Demographics and baseline data of the whole cohort (n 754 patients), of the subgroup of patients survived with a follow-up less than 7 months (Subgroup A–n 124) and the subgroup of patients survived with a follow-up more than or equal to 7 months and up to 60 months (Subgroup B–n 472)

	All patients (n 754)	Subgroup A (n 124)	Subgroup B (n 472)
Age	68 ± 14 years	72 ± 13 years	68 ± 15 years
Female	32% (n 239)	35% (n 43)	32% (n 149)
Male	68% (n 515)	65% (n 81)	68% (n 323)
Late referral	–	9% (n 11)	–
Age groups			
≤ 74 years	60% (n 451)	50% (n 62)	57% (n 271)
≥ 75 years	40% (n 303)	50% (n 62)	43% (n 201)
Cause of renal disease			
Diabetic nephropathy	16% (n 117)	15% (n 19)	15% (n 70)
Renovascular	24% (n 184)	27% (n 33)	26% (n 123)
Glomerulonephritis	15% (111)	24% (n 30)	17% (n 81)
Interstitial nephritis	3% (n 19)	0.8% (n 1)	1% (n 6)
Polycystic kidney disease	5% (n 39)	3% (n 4)	5% (n 25)
Unknown/other	37% (n 285)	29.8% (n 37)	35% (n 167)
Co-morbidities			
Diabetes	35% (n 263)	40% (n 50)	35% (n 164)
Coronary artery disease	40% (n 301)	46% (n 57)	38% (n 179)
Severe cardiomyopathy	38% (n 289)	46% (n 57)	37% (n 173)
Vascular disease	60% (n 452)	63% (n 78)	56% (n 267)
Malignancy	29% (n 218)	33% (n 41)	28% (n 131)
Vascular Access			
Arteriovenous fistula	35% (n 254)	14% (n 18)	36% (n 171)
Arteriovenous graft	2% (n 15)	3% (n 4)	1% (n 5)
Temporary catheter	57% (n 429)	76% (n 94)	54% (n 254)
Tunnelled catheter	7% (n 56)	6% (n 8)	9% (n 42)

Continuous variables are reported as mean ± standard deviation

### Predictors of outcome and Survival analysis

In the entire population, vascular disease (OR, 3.38; *p* = 0.000), malignancy (OR, 3.03; *p* = 0.000), severe cardiomyopathy (OR, 2.24; *p* = 0.000) and coronary artery disease (OR, 1.70; *p* = 0.004) were confirmed as independent factors predicting death. Moreover, all age classes (qualitative variable) were found to be a significant risk factor (Table 2). Regarding vascular access type at the beginning of haemodialysis, the adjusted OR highlighted tunnelled catheters as a significant protective variable (OR, 0.43; *p* = 0.017) compared to arteriovenous fistulas (Table 2). During the follow-up period (mean 39 ± 37 months, median 29), 59% (447/754) of patients died; at the time of data analysis 20% (152/754) were still alive with a median of previous haemodialysis treatment of 30 months (mean 44 ± 43). Cumulative survival according to Kaplan–Meier analysis and the incidence rate of death calculated as unadjusted HR showed a significantly higher risk of death in patients affected by the comorbidities analyzed in this study and among those who started the treatment with temporary catheters (HR 1.70; *p* = 0.000); adjusted HR confirmed this result (Table 3). Tunnelled catheters were not significantly associated with mortality.

**Table 2:** Results of the simple univariate and multivariate logistic regression model of the whole cohort (n 754 patients), of the subgroup of patients survived with a follow-up less than 7 months (Subgroup A–n 124) and the subgroup of patients with a follow-up survived more than or equal to or 7 months and up to 60 months (Subgroup B–n 472)

Variable	All patients (n 754)		Subgroup A (n 124)		Subgroup B (n 472)	
	Univariate	Multivariate	Univariate	Multivariate	Univariate	Multivariate
75–79 years old	3.20 (2–5)	1.9 (1.2–3.1)	–	–	4.1 (2.4–7.2)	2.0 (1.1–3.9)
80–84 years old	2.95 (1.9–4.5)	2.1 (1.3–3.5)	3.4 (1.3–9.2)	–	3.51 (2.1–5.9)	2.7 (1.5–5.0)
≥ 85 years old	5.21 (2.4–11.4)	3.4 (1.5–8)	3.7 (1.1–12.6)	–	7.8 (2.6–23.0)	5.4 (1.6–17.7)
Diabetes	1.56 (1.1–2.1)	**–**	–	–	1.6 (1.1–2.3)	**–**
Coronary disease	2.77 (2.0–3.8)	1.7 (1.2–2.4)	3.1 (1.4–6.5)	–	3.7 (2.4–5.6)	2.2 (1.4–3.6)
Cardiomyopathy	3.34 (2.4–4.6)	2.2 (1.5–3.2)	4.9 (2.2–10.9)	4.5 (1.8–10.5)	3.1 (2.7–4.7)	2 (1.2–3.2)
Vascular disease	4.49 (3.3–6.1)	3.4 (2.4–4.8)	4 (1.8–8.6)	3.2 (1.4–7.7)	5.6 (3.8–8.4)	4.2 (2.7–6.7)
Malignancy	3.2 (2.2–4.6)	3.0 (2.0–4.5)	3.5 (1.5–8.2)	4.6 (1.7–12.0)	3.4 (2.1–5.4)	2.9 (1.7–5)
Temporary catheter	1.72 (1.2–2.4)	**–**	–	–	2 (1.3–2.9)	**–**
Tunnelled catheter	–	0.43 (0.2–0.9)	–	–	–	0.26 (0.1–0.6)

Variables not statistically significant for either model are not included. Odds Ratios (ORs) are reported with their respective confidence intervals of confidence at 95% (95% CI)

**Table 3:** Results of the univariate and multivariate Cox proportional hazard regression model of the whole cohort (n 754 patients), of the subgroup of patients survived with a follow-up less than 7 months (Subgroup A–n 124) and the subgroup of patients survived with a follow-up equal to or greater than 7 months and up to 60 months (Subgroup B–n 472)

Variable	All patients (n 754)		Subgroup A (n 124)		Subgroup B (n 472)	
	Univariate	Multivariate	Univariate	Multivariate	Univariate	Multivariate
75–79 years old	2.2 (1.7–2.8)	1.8 (1.4–2.4)	–	**–**	2.4 (1.7–3.2)	1.9 (1.4–2.6)
80–84 years old	2.6 (2.1–3.4)	2.5 (1.9–3.2)	2.2 (1.2–3.9)	**–**	1.7 (1.3–2.3)	1.5 (1.1–2)
≥ 85 years old	4.2 (2.9–5.9)	3.5 (2.4–5)	2.5 (1.3–4.9)	**–**	1.8 (1.2–2.8)	**–**
Late referral			2.3 (1.1–4.8)	3.8 (1.7–8.4)		
Diabetes	1.5 (1.1–1.8)	1.3 (1.0–1.6)	**–**	**–**	1.3 (1.0–1.7)	**–**
Coronary disease	1.5 (1.3–1.8)	1.2 (1.0–1.5)	1.8 (1.1–2.8)	**–**	1.5 (1.2–2)	**–**
Cardiomyopathy	1.78 (1.5–2.1)	1.4 (1.2–1.7)	2 (1.2–3.2)	2.3 (1.4–3.7)	1.7 (1.3–2.1)	1.3 (1.0–1.7)
Vascular disease	1.8 (1.5–2.3)	1.4 (1.1–1.7)	1.8 (1–3)	**–**	2.2 (1.7–2.9)	1.9 (1.4–2.5)
Malignancy	1.5 (1.2–1.8)	1.4 (1.2–1.7)	1.7 (1.1–2.6)	1.9 (1.2–3.0)	1.5 (1.2–2)	**–**
Temporary catheter	1.7 (1.4–2.1)	1.5 (1.2–1.9)	**–**	**-**	1.6 (1.3–2.1)	1.4 (1.1–1.9)

Variables not statistically significant for either model are not included. Hazard ratios (HRs) are reported with their respective intervals of confidence at 95% (95% CI)

### Subgroup analyses

**Fig. 1:**
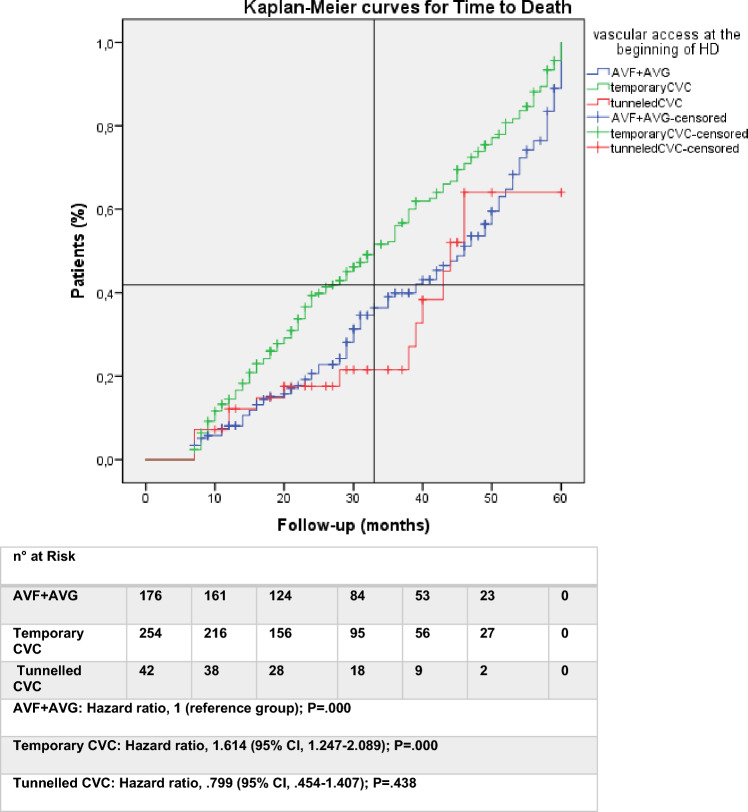
Time-to-death survival curves of patients between 7–60 months of follow-up (*n* = 472 patients, *n* = 271 events) depending on the type of vascular access at the start of haemodialysis. *AVF* arteriovenous fistula; *AVG* arteriovenous graft; *temporary CVC* temporary central venous catheter, *tunnelled CVC* tunnelled central venous catheter

We performed a subgroup analysis on patients (subgroup A–n 124) with a follow-up < 7 months to analyse the short-term data. During this time, 60% (74/124) of patients died, of whom 61% (45/74) were classified as elderly and 15% (11/74) started haemodialysis without proper planning (late referral). Among the deceased, 81% (60/74) started treatment with a catheter. Malignancy (OR, 4.57; *p* = 0.002), severe cardiomyopathy (OR, 4.35; *p* = 0.001), and vascular disease (OR, 3.22; *p* = 0.008) were all found to be statistically significant risk factors (Table 2). In the survival analysis, severe cardiomyopathy (HR, 2.28; *p* = 0.001) and malignancy (HR, 1.89; *p* = 0.008) were significant risk factors, but the strongest association was found to be late referral at the start of haemodialysis (HR, 3.79; *p* = 0.001). Age class and vascular access were not statistically significant (Table 3). Heart failure or fatal myocardial infarction, severe vascular disease or cerebrovascular events were the leading causes of death in 51% (38/74), whereas septic events caused 23% (17/74) of the events. A second subgroup analysis was performed on patients (subgroup B–n 472) with at least 7 months of follow-up and a maximum of 60 months (median follow-up 27 months; mean 29 ± 15, min–max 7–60) from the start of maintenance dialysis (Fig. 1). Fifty-seven percent (271/472) of patients died within this term. Interestingly, the adjusted OR of using tunnelled catheter as vascular access yielded higher protection against death (OR 0.26, *p* = 0.002) than arteriovenous fistula. Vascular disease, on the other hand, was confirmed as the strongest predictor of death (OR, 4.20; *p* = 0.000). Vascular disease was found to be the strongest risk factor (HR, 1.90; *p* = 0.000) in the survival analysis as well. Furthermore, in the subgroup of elderly patients, the use of tunnelled catheters emerged as a predictive factor (OR 0.25, *p* = 0.021) against the use of arteriovenous fistulas or grafts. Of these patients, 56% (153/201) died during follow-up; peripheral arteriopathy accounted for 37% (57/153) of deaths and sepsis complicated 12% (19/153) of cases. Cardiovascular disease and malnutrition-dialysis cachexia were the causes of death in 19% (29/153) in both conditions.

## Discussion

The results that were observed in our study confirm a trend that has emerged in the past few years [18–20]. In fact, the incidence of elderly patients in our cohort more than doubled over the last two decades (from 17% in 2001 to 42% in 2020), representing 40% of the total. Comorbidities at the beginning of haemodialysis were common, with vasculopathy being the most represented (60% of patients). With regard to vascular access, we report a prevalence of catheters (temporary plus tunnelled) higher than the reported average (64%), and of all catheters in use at the end of follow-up 53% (174/329) were being used in elderly patients. Of note, the high prevalence of temporary catheters observed at the end of the follow-up period might be related to the need for an alternative vascular access in hospitalised or critical patients. As early as 1996, several studies reported a worldwide increase in the use of catheters during maintenance haemodialysis [3, 18–21]. Regarding predictors of death, our data yielded a strong association with mortality in the univariate analysis (uOR 1.72; *p* = 0.001) for temporary catheters, whereas no association was found for tunnelled catheters compared to arteriovenous fistulas. Patient survival and its association with type of vascular access has been widely discussed [3–5, 15]: in Europe, among patients who started renal replacement treatment between 2005 and 2009, 2-year survival was 82% vs 69% for patients with fistulas vs catheters (both tunnelled and temporary), respectively [18]. We found similar 2-year survival: 83% vs 62% in patients with a fistula vs catheter, both tunnelled or temporary. However, taken individually, the 2-year survival was 60% vs 77% for temporary vs tunnelled catheters. Time-to-death analysis estimated the incidence rate of death to be significantly higher in patients with temporary catheters (HR 1.70) compared with fistulas, but after adjustment for confounding factors this incident rate of death decreased from 70 to 54% (HR 1.54; *p* = 0.000); the adjusted HR of tunnelled catheters was not associated with the risk of death. The significant decrease in the HR after correction for confounding factors can point to how much the choice of vascular access may be influenced in the clinical setting by several issues, such as age and comorbidities [24]. Moreover, since in the multivariable analysis having a tunnelled catheter was associated with a lower risk of death than having an arteriovenous fistula, one might speculate that the choice of the vascular access only has a limited impact on patient survival, more driven by comorbidities and age, which can impact on fistula maturation [14, 24]. In line with this, the use of arteriovenous fistulas might expose elderly or fragile patients to cardiovascular events (e.g. heart failure), which might be avoided by using catheters.

Patient survival in haemodialysis, especially with respect to risk of death and the use of catheters as vascular access, has been widely debated [3, 7, 14, 24]. In our study, neither vascular access type nor age emerged as predictor of mortality, suggesting that health status before starting maintenance haemodialysis might account for an important part of the risk previously attributed to catheter use [2, 13]. Furthermore, survival analysis showed that the impact of vascular disease (HR, 1.9; *p* = 0.000) and age class was greater than that of having a temporary catheter, while arteriovenous fistulas and tunnelled catheter did not impact on mortality. Among the strengths of the study, the length of follow-up and the homogeneity of the treatments minimize the risk of bias among our cohort; on the other hand, given the observational nature of the study, the results should be interpreted with caution, as all observations from a real-life setting.

In conclusion, our single-centre long-term experience confirms that the choice of vascular access is a complex medical decision that requires a holistic approach, and suggests that mortality is more strictly associated with comorbidities than with the type of vascular access.

## Data Availability

The dataset generated during and/or generated during the study are available from the corresponding author on reasonable request
